# Polar-Net: Green fruit instance segmentation in complex orchard environment

**DOI:** 10.3389/fpls.2022.1054007

**Published:** 2022-12-14

**Authors:** Weikuan Jia, Jie Liu, Yuqi Lu, Qiaolian Liu, Ting Zhang, Xishang Dong

**Affiliations:** ^1^ School of Information Science and Engineering, Zaozhuang University, Zaozhuang, China; ^2^ School of Information Science and Engineering, Shandong Normal University, Jinan, China; ^3^ Key Laboratory of Facility Agriculture Measurement and Control Technology and Equipment of Machinery Industry, Jiangsu University, Zhenjiang, China

**Keywords:** homo-chromatic fruit, Polar-Net, DenseNet, instance segmentation, green fruit

## Abstract

High-quality orchard picking has become a new trend, and achieving the picking of homogeneous fruit is a huge challenge for picking robots. Based on the premise of improving picking efficiency of homo-chromatic fruit in complex environments, this paper proposes a novel homo-chromatic fruit segmentation model under Polar-Net. The model uses Densely Connected Convolutional Networks (DenseNet) as the backbone network, Feature Pyramid Network (FPN) and Cross Feature Network (CFN) to achieve feature extraction and feature discrimination for images of different scales, regions of interest are drawn with the help of Region Proposal Network (RPN), and regression is performed between the features of different layers. In the result prediction part, polar coordinate modeling is performed based on the extracted image features, and the instance segmentation problem is reduced to predict the instance contour for instance center classification and dense distance regression. Experimental results demonstrate that the method effectively improves the segmentation accuracy of homo-chromatic objects and has the characteristics of simplicity and efficiency. The new method has improved the accuracy of segmentation of homo-chromatic objects for picking robots and also provides a reference for segmentation of other fruit and vegetables.

## 1 Introduction

At present, among the various aspects of fruit and vegetable production management, yield prediction and picking operations are important supports to realize their scientific management, but at present, they are still mainly manual operations, presenting such phenomena as high labor, high production costs, and low operational efficiency. As the population ages and urbanization intensifies, agricultural labor is increasingly scarce, bringing many difficulties to agricultural production. In order to alleviate the above-mentioned difficulties in fruit and vegetable production and improve the competitiveness of the product market, it is necessary to improve the automation and intelligent operation level of fruit and vegetable production management. Accurate segmentation and identification of target fruits is the basic guarantee for fruit and vegetable yield prediction and automatic picking operations (Witus et al., 2018; Fu et al., 2020; Jia et al., 2020; Tang et al., 2020). At present, in the complex unstructured actual orchard environment, the interference of various factors such as light, branch and leaf shading, and fruit color poses a great challenge for the accurate segmentation of target fruits. Exploring efficient and accurate segmentation algorithms for green target fruit, reducing the rate of missed and misidentified green fruit, improving the recognition accuracy of target fruit, and helping scientific management of fruit and vegetable production has attracted the attention of many scholars.

Traditional machine learning theory and optimal algorithm have played an important role in the field of image segmentation (Ilea and Whelan, 2011; Sharma and Suji, 2016; Li et al., 2020; Li et al., 2022), and many scholars have conducted in-depth research on the accurate segmentation and recognition of green target fruits with the help of machine learning, and promising results have been achieved in both spherical fruit segmentation and long fruit segmentation. Li (Li et al., 2016) proposed a fusion of Fast Normalized Cross Correlation (FNCC) (Li et al., 2020) and Circular Hough transform (CHT) (Pedersen, 2007) method to detect immature green citrus with a success rate of 84.4%. FNCC and CHT methods to detect immature green citrus, which can effectively reduce false positive detection, and the success rate of the new method reached 84.4% to establish an initial yield localization system in orchards. Arefi (Arefi et al., 2011) proposed segmentation algorithm using a machine vision system knowing robot picking arm for picking ripe tomatoes, the adopted algorithm extracts ripe tomatoes by removing the background from RGB images and locates ripe tomatoes using morphological features of the images with 96.36% accuracy. Huang (Huang et al., 2018) used a graph segmentation algorithm to segment green peach images into multiple layers, calculate the saliency map of each layer and fuse them to obtain discriminative regional feature integration (DRFI) saliency map, which reduced the miscommunication of low probability fruits with 88.3% correct recognition rate. Wang (Wang et al., 2021) proposed a new kernel density estimation optimized clustering segmentation algorithm kernel density clustering (KDC), for the segmentation problem of orchards affected by complex environments, which significantly reduces the computational complexity by simple iterative clustering simple linear iterative clustering (SLIC) (Noh and Woodward, 1976) algorithm. It achieves efficient segmentation of target images. Most of the above methods use features based on color, texture, and shape as the basis for target fruit segmentation, however, these features are easily disturbed by light and environmental changes, resulting in color distortion or weaker texture features; branch and leaf occlusion or overlap also easily lead to the absence of target fruit shape features; and the color characterization ability of green target fruit is reduced by background color interference. All these factors affect the segmentation, identification and positioning accuracy of the target fruit, and it is difficult to meet the operational requirements of various intelligent devices.

With the gradual entry of deep learning, more and more computer vision problems are beginning to be solved with the help of neural network architecture. The end-to-end processing of deep learning has achieved better results in the direction of image processing, thus drawing attention to agriculture, and better progress has been made with the help of deep learning to solve the problem of leaf homo-chromatic system, using the advantages of end-to-end automatic detection process and deep extraction of image features, eliminating many complex operations of traditional vision algorithms, and the accuracy has been significantly improved, and the segmentation speed has been somewhat improvement. Li (Li et al., 2021) proposed a segmentation model U-Net (Ronneberger et al., 2015) suitable for small sample datasets, merged edge features and high-level features of U-Net with the help of Atrous Spatial Pyramid Pooling (ASPP) (Sullivan and Lu, 2007) structure, combined with ASPP to achieve the acquisition of boundary semantic information of the target image, as well as to resolve the resolution of the feature map and the receiving field to achieve accurate segmentation of fruits in the target image. Jia (Jia et al., 2021) proposed a robust segmentation model RS-Net developed specifically for fruit production, which extended Mask R-CNN (He et al., 2017) by embedding an attention mechanism to focus more on informative pixels and suppress the segmentation disadvantages caused by natural environments such as occlusion and lighting, thus achieving accurate and effective recognition and segmentation of apples in natural orchards. Liu (Liu et al., 2021) proposed a DLNet segmentation model for the severe occlusion problem in complex orchard environments, which extends the fully convolutional one-stage (FCOS) (Tian et al., 2019) model by embedding a double-layer graph attention networks (GAT) (Veličković et al., 2017) structure and attention mechanism to segment the occluded and occluded objects with the help of the GAT structure to achieve accurate detection and segmentation of the occluded region. Sa (Sa et al., 2016) proposed two feature extraction algorithms, multi-scale multilayer perceptron and convolutional neural network, which were used for apple segmentation and localization, but knowledge was identified for a single class of fruit of the same color family. Among the above fruit recognition algorithms designed based on CNNs, they outperformed traditional machine learning vision methods in several dimensions. However, these methods rely on setting predefined anchor frames to the original image during detection and then adjusting the network to get the bounding box, which can be seen that these methods require high computational and storage resources, slow recognition speed, poor real-time, and stability and power consumption during normal operation are not guaranteed, which cannot yet be directly applied to fruit and vegetable picking equipment.

After analyzing the above domestic and international status quo, this paper proposes a Polar-Net segmentation model to achieve accurate segmentation of homochromatic fruits, which is an anchor-frameless, single-stage, fully convolutional approach to compress the model capacity and reduce computational and storage resources without losing the accuracy of anchor-frame-based target detection. This method can accelerate the training and detection speed of the model, which solves the contradiction between accuracy and speed in previous fruit recognition and detection methods, and takes good care of the detection accuracy of green fruits and their working efficiency. In addition, to better adapt to the complex orchard environment where the persimmon dataset in this paper is located, the original PolarMask (Xie et al., 2020) model is therefore improved based on its backbone network by replacing the Residual Network (ResNet) (He et al., 2016) in the backbone network with the Densely Connected Convolutional Networks (DenseNet) (Huang et al., 2017) to reduce the computation; Polar-Net embeds the Criss-Cross Attention (CCA) module (Huang et al., 2019) in Feature Pyramid Network (FPN) (Lin et al., 2017a) to fuse into Cross Feature Network (CFN) so that it focuses on local The model incorporates RegionProposal Network (RPN) (Girshick, 2015) in the feature extraction stage to optimize the extraction of Region Of Interest (ROI) regions for subsequent accurate segmentation and improve the model’s detection accuracy under the interference of leaf occlusion, overlap, and background of the same color. The Polar-Net model can meet the multiple requirements of speed, accuracy, and robustness in applying various intelligent technologies.

## 2 Data collection and dataset creation

### 2.1 Data collection

**Figure 1:**
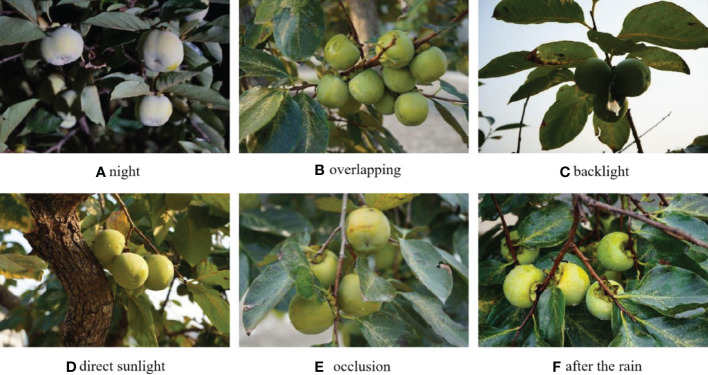
Images of green target fruits of persimmon under different environments (**(A-F)** are listed for green fruits at various angles and under different light).

In this study, the immature persimmon is used as the research object, which fits the characteristics of green spherical fruit. The accurate segmentation of the target fruit is very challenging due to the influence of light and branch background. The acquired image objects are immature persimmons (green), and the varieties are oxheart persimmon, chicken heart yellow, and mirror persimmon. The image acquisition locations were the back mountains of Shandong Normal University (Changqing Lake Campus) and the southern mountains of Jinan City. Canon EOS 80D SLR camera was used for image acquisition, and the camera used CMOS (complementary metal oxide semiconductor) image sensor. The image resolution was 6000 pixels × 4000 pixels, saved as JPG format, 24-bit color image. Considering the operational scenarios of orchard yield measurement and automatic machine picking, the image acquisition was as comprehensive as possible, simulating different camera positions and multiple angles around the fruit trees to obtain images of the target fruit in different poses. The acquisition time includes multiple time periods during the day and night, where the daytime acquisition is a natural light environment (including the case of downlight and backlight) and the night acquisition uses LED as an auxiliary light source. During the acquisition process, different time periods (with different light intensities), different weather, different light angles, and different camera angles should also be considered, i.e., the complex actual operating environment of the orchard. A total of 568 images of green persimmons under different environments were collected, as shown in **Figure 1**, including nighttime, overlapping, backlighting, down-lighting, shading, after rain, and many other situations.

From **Figure 1**, it can be seen that the target fruit boundary is not clear in the branch-obscured or fruit-overlapping images; the target fruit surface is weakly lit in the backlit images, making the segmentation boundary of the fruit not obvious, and even the naked eye is difficult to identify its boundary; for the images with shadows or raindrops, the segmentation accuracy of the target fruit is also affected; the images at night are partially noisy due to the influence of the acquisition environment, making segmentation more difficult. In addition, the similar color of the target fruit and the background will also bring some difficulty to the segmentation.

The collected image samples maximally considered the complex actual environment of the orchard, and 568 images were sorted and classified, including overlapping, parasite, after rain, backlight, nighttime, same color system, different color system overlapping, etc. The detailed information of the sample distribution is shown in **Table 1**.

**Table 1:** Sample distribution details of green fruit images (The following table lists the number of persimmons in the dataset under different scenarios).

Conditions	overlapping	Direct sunlight	After the rain	Backlight	Night	Cover	No occlusion overlap
Number of persimmon images	523	109	113	207	125	518	98

### 2.2 Data set production

To meet the requirements of real-time detection of orchard targets, the image resolution is compressed and processed from 6000×4000 to 600×400, which significantly reduces the computational volume of the model without affecting the segmentation accuracy. To further improve the image quality, the images are scaled, randomly flipped and normalized.

The dataset was obtained using LabelMe software, labeling the target fruit to produce a COCO format (Lin et al., 2014) dataset. The edge contours of the target fruit were labeled using LabelMe using labeling points and given labeling labels. The labeling points divide the image into two parts, with the green target fruit inside the labeling points and the rest as the background. All the labeling information such as label, label point coordinates, etc. will be saved in a json file corresponding to the original image. After that, the json file will be converted into COCO format dataset using LabelMe. After the dataset is created, it is divided into training establish and testing set, where the number of images in the training set is 398 and the number of images in the test set is 170.

## 3 Polar-Net: Same color fruit instance segmentation model

**Figure 2:**
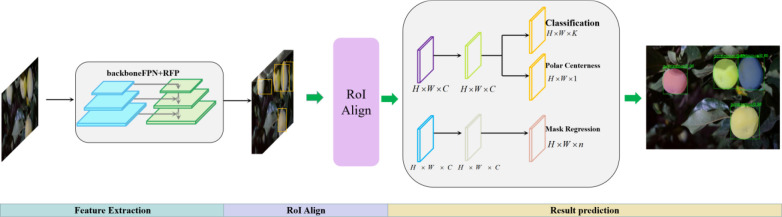
Polar-Net structure diagram (including three parts of feature extraction, ROI Align, and result prediction).

To improve the segmentation accuracy and speed of green target fruit in a complex real orchard environment, a fast and efficient Polar-Net instance segmentation model is proposed in this study. The network framework of this model uses Densely Connected Convolutional Networks(DenseNet) as the backbone network for feature extraction, introduces the FPN structure to adapt to the multi-scale variation of feature extraction, and adds CCA fusion to CFN on top of FPN to achieve feature extraction of insignificant regions, and subsequently adds the RPN structure to achieve feature discrimination to realize accurate extraction of fruit features. The extracted features are output to the head network and enter the parallel classification and regression branches to realize the construction of the poles and pole coordinates of the fruit according to the extracted features and output the final segmented instance contour. The overall network structure of the original model is shown in **Figure 2**.

### 3.1 Backbone

The Polar-Net model selects DenseNet as the backbone network for the initial extraction of image features, and outputs the feature map from the dense block, whose feature maps are fused according to the top-down and lateral connection, thus making the deep feature map and the shallow feature map have the same level of semantic capacity to obtain the feature pyramid FPN. Introducing FPN, realizing the extraction of multi-scale The FPN is introduced to achieve the extraction of multi-scale and multi-level features, thus satisfying the extraction of small feature areas caused by the small green fruit areas due to external factors such as lighting and shading.

#### 3.1.1 DenseNet

**Figure 3:**
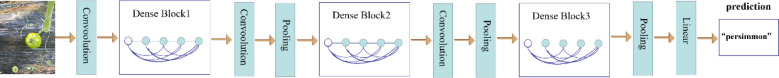
DenseNet structure diagram (including three dense block blocks, three convolution and pooling operations).

DenseNet is a network that starts from features and aims to use the least number of parameters to see more feature information. Compared with ResNet, DenseNet improves feature reusability by one level, and each layer uses the summation of feature maps from all previous layers as input, which in turn greatly enhances feature transmission and encourages feature reuse. Dense blocks ensure that low-dimensional features are not completely discarded and can enhance the sensitivity to small target regions. The output of each cell establishes a short connection with the output of each convolutional layer of the next cell, thus achieving continuous transmission of information, and the specific network structure is shown in **Figure 3**.

For a convolutional network, assuming an input of *x*_0_ to the image. The network is composed of L layers. Each layer implements a nonlinear transformation *H_i_* (.), where i denotes the *i_th_* layer, and then what the *i_th_* layer obtains is a feature mapping map *x*_0_, *x*_1_, *x*_2_,…*x_i_*_-1_ of all previous layers as input


(1) x =1H1[x0,x1,x2,…,xi−1]

Here, *F_d_*_-1_ denotes the cascade of feature mappings. Defining the input and output of the *d_th_* layer as *F_d_* and *F_d_*_-1_, respectively, and the number of feature mappings as *G*_0,_ Since the connection between the input side of the cell and the convolution layer is used, it is necessary to compress the feature map at the end of the cell, and therefore the number of feature maps is controlled. The convolution by 1×1 will be represented when:


(2) $$F_{d,LF}=H_{LFF}^{d}\left\{F_{d-1},F_{d,1},\ldots F_{d,c}\right\}$$

the output of the *c_th_* convolutional layer can be expressed as:


(3) $$F_{d,c}=H\left[F_{d-1},F_{d,1},\ldots F_{d,c-1}\right]$$

Thus the final output unit will be expressed as:


(4) $$F_{d}=F_{d-1}+F_{d,LF}$$

In this study, DenseNet is set up with three dense blocks, each of which contains a 3×3 convolutional structure. In order to decrease the number of input feature maps, a transition layer is added in front of each dense block, aiming at both dimensionality reduction to reduce the computation and fusion of the features of each channel. The overlayer is set at a 1×1 convolutional layer, the number of channels is 4k, k is the growth rate, and the growth rate 4k means that the dimensionality of the output feature map of each dense layer is four. To ensure the maximum flow of information between each layer, all network layers are directly related. The input of each layer is the sum of the mapped outputs of the previous layer, and its peculiar feature mapping results are also used as the input of the subsequent layers to further maintain the feedforward features of the network and achieve the reuse of features.

#### 3.1.2 FPN

Although the final output feature map of DenseNet network contains rich semantic information, its resolution is very low after continuous up sampling, and detail information such as boundary is basically lost, so it is suitable for extracting large scale target features. While in the complex orchard environment, the interference of external factors such as shading and lighting makes the target fruit area small, in order to achieve feature extraction for small area fruits, FPN is introduced. FPN is used to solve the multi-scale prediction problem by fusing the semantic information of the deep feature map and the detail information of the shallow feature map to construct a feature pyramid, and distributing the targets to be detected at different scales to the features at different levels of the pyramid graphs responsible for prediction. By applying FPN to fruit recognition, it can effectively improve the detection effect of the model for different scales, especially small-scale target fruit, and thus can better guide the recognition picking and path planning of fruit and vegetable picking robots.

**Figure 4:**
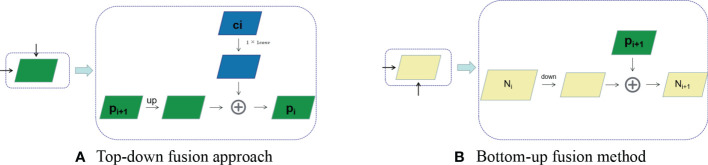
FPN connection method (*p_i_* denotes the output of each layer, the income of both **(A)** and **(B)** plots are the output of DenseNet network).

In this paper, the feature maps *C*_1_, *C*_2_, *C*_3_ output from the three dense blocks in DenseNet are used to construct the feature pyramid. *C*_1_, *C*_2_, *C*_3_ are fused by 1×1 convolutional lateral connections with 2-fold up-top-winning top-down connections to obtain *P*_1_, *P*_2_, *P*_3_, as shown in **Figure 4A**. Then *P*_4_, *P*_5_ is obtained by *P*_3_ after two down sampling, as shown in **Figure 4B**, to obtain the constructed feature pyramid {*P*_1_, *P*_2_, *P*_3_, *P*_4_, *P*_5_}.

The features extracted by the DenseNet network will lead to errors in the localization information of the deep layer network after multiple down-sampling and up-sampling operations, and the low layer network can provide more accurate feature location information. FPN constructs feature pyramids with deeper semantic information through top-down and lateral connection connections, and accumulates the low layer features and high layer features, making the FPN structure can fuse the shallow layer with high resolution and the deep layer with rich semantic information, enabling the construction of a feature pyramid from a single input image at a single scale to a feature pyramid with strong semantic information at all scales quickly, without incurring significant time and space costs.

#### 3.1.3 CFN

**Figure 5:**
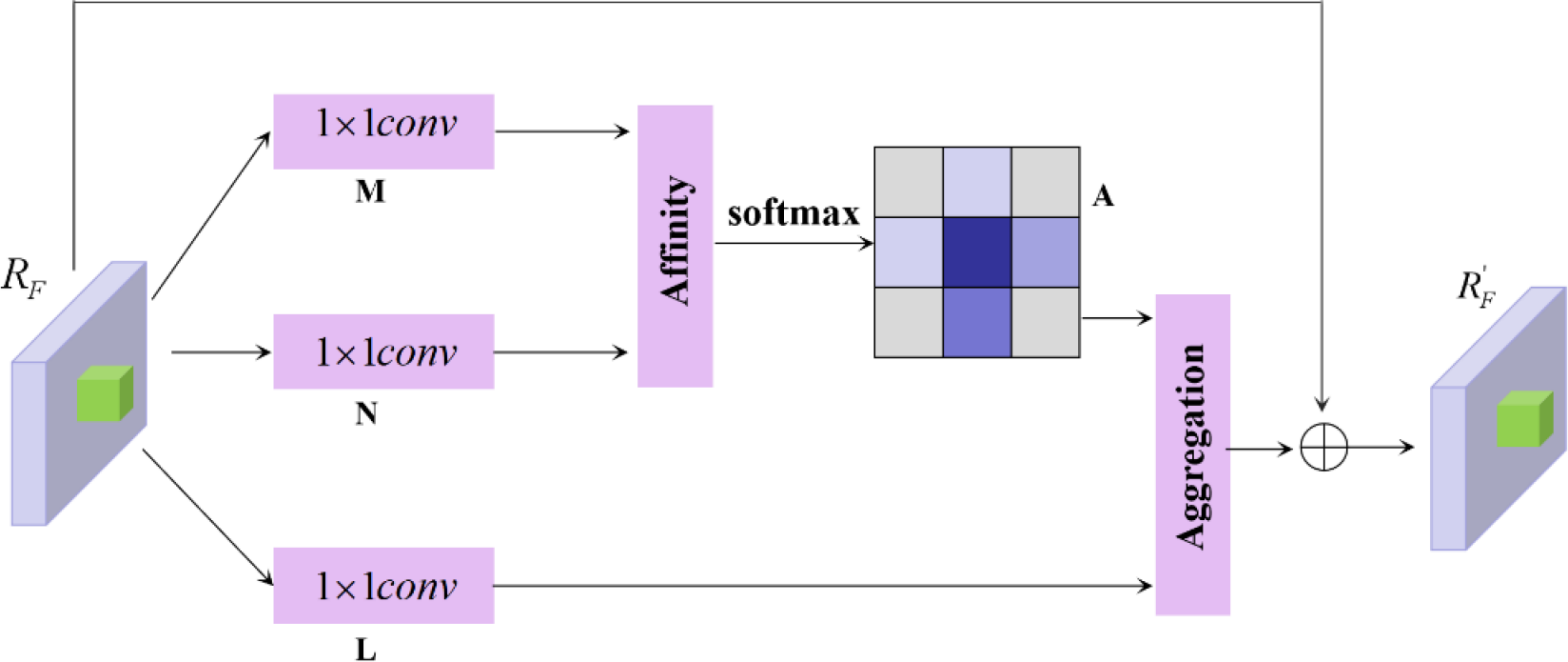
CCA module (*R_F_* feature maps are processed by the CCA module to highlight tabular features).

In the actual orchard environment, the target fruit area brought by irresistible factors (lighting, shading) is too small, and this small area will be continuously diluted with semantic feature information after the continuous up sampling operation of FPN, which increases the difficulty of target fruit feature extraction. In addition, the extracted features should be the balanced information of semantic features on each image, and after the FPN structure will make more focus on the feature information of adjacent information, so that the semantic feature information of non-adjacent level will be continuously diluted in each fusion. To alleviate the above problem, the Criss-Cross Attentio (CCA) module is proposed to be embedded in the FPN to fuse into a balanced feature pyramid CFN, which uses lightweight computation and memory to model the contextual relevance of local feature processes with the help of attention mechanism, and the CCA module collects contextual feature information in horizontal and vertical directions to enhance the representation of pixel features. Thereby highlighting epistemic features, the structure of which is shown in **Figure 5** below.

After the feature map F obtained by DensNet + FPN network extraction, the convolutional layer is first used to obtain the reduced-dimensional feature map *R_F_*, and the *R_F_* feedback is embedded into the CCA module. Space size of *R_F_* is *C* × *H* × *W*. After entering CCA, the feature maps M and N are generated after two 1×1 onvolutions, and their space size is *C’* × *H* × W; M and N are calculated by Affinity operation to obtain L, and the space size of L is (*W* × *H*-1) × *W* × *H*. The SoftMax operation is performed on the dimension of L to obtain A, and the space size of A is still (*W* × *H*-1) × *W* × *H*. For X at any position in the feature map, the features of *C’* channels are extracted and obtained to form the feature map at that position *C_x_*, the size is 1×1×*C’*, and a similar operation is performed to extract the feature vector at the cross position, noted as *Ω_x_*, there are *H*+*W*-1 positions, so the vector size is (*H*+*W*-1) × *C’*.

*R_F_* after 1×1 convolutions of to obtain *R’*_F_, *Ω* of size *C*×*H*×*W*, define the feature vector *V_x_* of size 1×1×*C’*, obtain the cross feature vector *Ω_x_* of size (H+W-1)×*C’* at position x, and perform aggregation to obtain *R_F_*,*R_F_* of space size *C*×*H*×*W*.


(5) $$R{'}_{F}=\sum_{i\in \left|\Omega x\right|}Z_{i,x}\Omega_{i,x}\;+RF$$

In Eq. 5, *Z_i,x_Ω_i,x_* is the position corresponding elements are multiplied by it and context information is added to the local features *R*_F_ to enhance the local features and to enhance the pixel-wise representation. The feature map *R’*_F_ is obtained after the CCA module to provide remote contextual information for each pixel in a crisscross manner, aggregating contextual information in horizontal and vertical directions. Thus, it makes a wide range of contextual views and selectively aggregates contextual information based on the spatial attention map.

#### 3.1.4 RPN regional generation network

**Figure 6:**
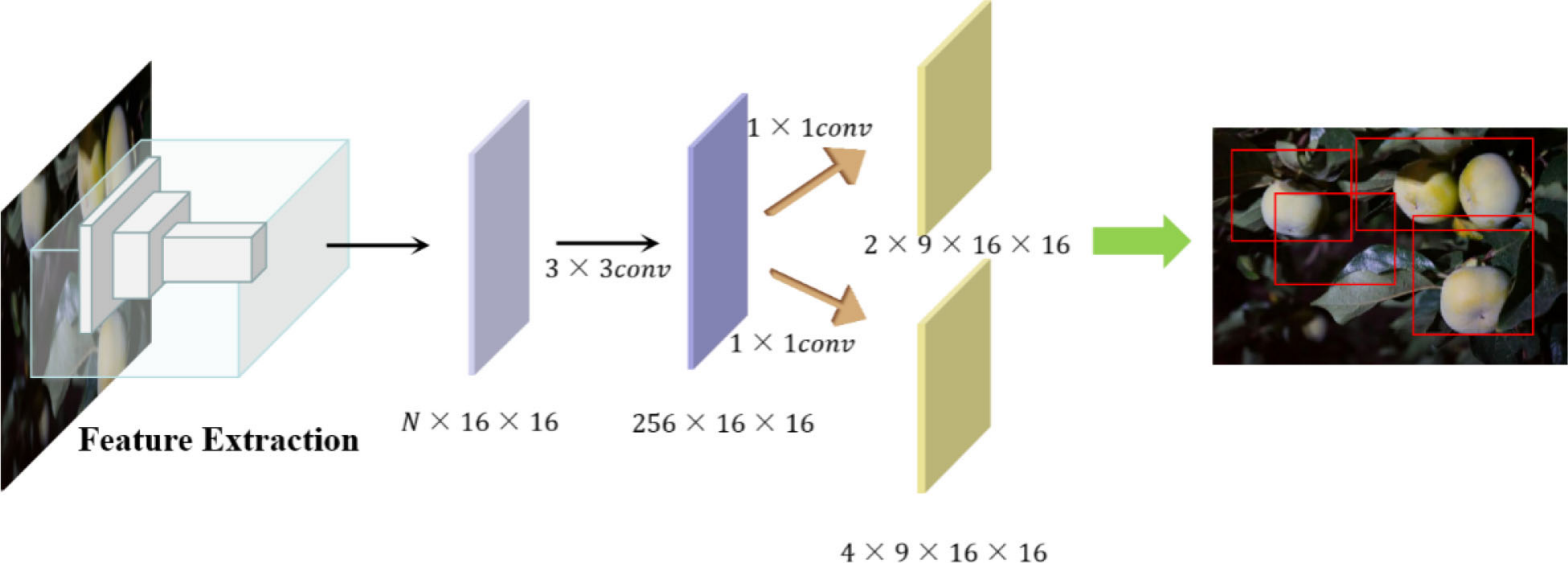
RPN structure workflow diagram (to generate the final effect of the candidate box for simple and beautiful, selected four candidate boxes).

After the DenseNet + FPN and CFN networks, the extracted feature information concerns all the information elements of the input image’s location, and the RPN is introduced to obtain the region of interest in the target fruit segmentation, whose structure is shown in **Figure 6**. Firstly, after a convolution of 3×3, a feature map of 256×16×16 is obtained, which can be regarded as 16×16 256-dimensional feature vectors, and then after two convolutions of 1×1, a feature map of 18×16×16 and a feature map of 36×16×16 are obtained respectively, that is, 16×16×9 results, each containing two scores and four coordinates, and then combined with the pre-defined anchor frame, after post-processing, the candidate region ROI is obtained.

In this paper, for the predefined anchor boxes, due to the large differences in the size of the target fruits in the images, as well as the overlap between the target fruits and the influence of branch and leaf occlusion factors, which make the horizontal and vertical ratios of the exposed area vary greatly, according to the image characteristics, three different area scales of 16×16, 64×64 and 128×128, three in the aspect ratios of 1:1, 1:2 and 2:1 are randomly combined, and the above 9 bounding boxes are used in the extracted 9 anchor boxes are generated on the extracted feature map. Finally, the feature candidate boxes are input into the classification branch of the head structure to get two scores, foreground (object) and background score, to judge the candidate box categories.

### 3.2 Result prediction

After obtaining the candidate frame of the target region, an efficient instance segmentation model based on Polar-Net green target fruit is further constructed, and the specific segmentation process is as follows: based on the given instance mask, the central sample (*x_cen_*, *y_cen_*) and the points on the instance contour are sampled based on the instance; the distance from the center of the sample to the points on the contour is predicted {*l*_1_, *l*_2_, *l*_3_,…,*l_n_*} and a ray of length {*l*_1_, *l*_2_, *l*_3_,…,*l_n_*} is drawn from the center of the sample at a fixed angle (here n=36).

Based on the points on the ray, the points on the contour are determined using NMS, and the specific contour of the instance is inscribed. The new method can obtain the center sample based on the pre-training of the network, and the outline of the instance is obtained by polar coordinate construction based on the predicted distance and the center sample, from which the problem is converted into a prediction that determines the distance to the outline point.

#### 3.2.1 Selection of the central sample

To obtain the masked contour of the target fruit, a polar coordinate system is established for the ROI region obtained through the above network, and the range of polar sitting system pole samples is selected first. According to the classification branch of Polar-Net model, the pixel regions in the four directions above and below the center of gravity are selected based on the center of gravity of the image, and a total of N~M pixel points are selected, and the selected pixel points are taken as the center samples. There are various choices for the selection of the center sample, and the center of gravity of the target fruit is selected as the center of gravity sample in this study.

#### 3.2.2 Prediction {*l*_1_, *l*_2_, *l*_3_,…,*l_n_*}

The polar coordinate system is established with the selected central sample as the center and the polar axis , with the specified length unit and clockwise as the positive direction; specifically, a ray is drawn from the center of the pole as the polar axis, one is selected as the length unit, and the polar coordinate system is established with the clockwise direction as the positive direction.

After the polar coordinate system is established, the distance from the center sample to the contour boundary {*l*_1_, *l*_2_, *l*_3_,…,*l_n_*} needs to be obtained to obtain the contour points of the green target fruit. First, a direction is selected from the center of polar coordinates, and then the selected direction is scanned outward pixel by pixel to determine whether it belongs to the target object, until all pixels of the whole picture are judged, and finally the farthest pixel point belonging to the object is selected as the length of the ray emitted from that direction by that point.

The number of rays is chosen, according to experiments, the more the number of rays will bring more gain, but too many will also cause saturation, because after the number reaches a certain level, it is close to the actual value. The best results were obtained through experimental studies when the number of rays was chosen to be 36. Therefore, when predicting the length, 36 rays are selected, and one predicted length is selected at 10° intervals.

#### 3.2.3 Center-ness

In this model, the centrality concept is introduced with the help of FCOS ideas, which is used to suppress some low-quality detection frames without introducing additional hypermutate. Unlike the FCOS idea, Polar-Net is concerned with instance segmentation under one stage rather than target detection. When predicting the result of a certain instance segmentation, a set of distances from the centroid of the contour can be obtained {*l*_1_, *l*_2_, *l*_3_,…,*l_n_*}. As a result, Polar Center-ness can be obtained as following.


(6) $$\text{Polar Center}-\text{ness }=\sqrt{\frac{min\left(\left\{l_{1},l_{2},\ldots ,l_{n}\right\}\right)}{max\left(\left\{l_{1},l_{2},\ldots ,l_{n}\right\}\right)}}$$

The network outputs two branches: classification and centrality, multiplying the above centrality with the number of classifications (k) to obtain the final confidence score, and then after setting the confidence score threshold to 0.05, assembling the instance profile based on the maximum 1k highest scoring predictions for each FPN level, merging the highest predictions for each level, and applying a non-maximum suppression (NMS) with a threshold of 0.5 to generate the final result.

#### 3.2.4 Contour masking

In the polar coordinate system, the specific coordinates of the points on the outline of the green target fruit are further obtained from the coordinates of the center point (*x_cen_*, *y_cen_*) and the set of 36 ray lengths {*l*_1_, *l*_2_, *l*_3_,…,*l_n_*}, so as to obtain the real outline image of the green fruit.

*x*_*a*_=*cos*∂_*a*_∗*l*_*a*_+*x*_*cen*_ (7)


(8) $$y_{a}=sin\partial_{a}*l_{a}+y_{cen}$$

Where, the obtained (*x*_a_, *y*_a_) is the coordinate point on the Perspex contour, *l*_a_ is the specific ray that the calculated coordinate point depends on, ∂_*a*_ is the angle of the offset of the center point of the with under the selected ray, and (*x_cen_*, *y_cen_*) is the selected center sample coordinate. The coordinates of the points on the contour are calculated according to the formula, and an arbitrary point is selected, and the instance contour is obtained by connecting these contour points in sequence along the clockwise direction, at an interval of 10° each time.

### 3.3 Loss function

The model is trained by iteratively predicting the loss between the prediction frame and the real frame after model training to achieve the green fruit segmentation ability, and updating the model training parameters according to the back propagation speed of the loss value until the loss is reduced and converges to a value interval to obtain an optimal loss function for model training. The loss function in Polar-Net model is based on the prediction target of each branch of the model, the task type, the proportion of positive and negative samples. The loss function consists of *L_cls_、 L_cen_、 L_mask_* three parts, *L_cls_、 L_cen_、 L_mask_* which are calculated by Focal Loss (Lin et al., 2017b), BCE Loss (De Boer et al., 2005), and IoU Loss (Yu et al., 2016), respectively, and its loss function is shown as follows.


(9) $$L=L_{cls}+L_{cen}+L_{mask}$$

Where the loss function of the classification branch *L_cls_* , and the loss function of the centrality branch *L_cen_* , is calculated as showed below.


(10) $$L_{cls}=\frac{1}{N_{pos}}\sum_{x,y}L_{cls}\left(cl_{x,y}cl_{x,y}^{*}\right)$$

(11) $$L_{cen}=\frac{\partial }{N_{pos}}\sum_{x,y}cl_{x,y}^{*}L_{cen}(cen_{x,y},cen_{x,y}^{*})$$

In Eq. *cl_x,y_*, *cen_x,y_* are the predicted values of classification branch and centrality branch at spatial position (*x, y*), respectively; $cl_{x,y}^{*},\;cen_{x,y}^{*}$ corresponds to the training value at spatial position (*x, y*); *N_pos_* denotes the number of positive samples; ∂ is the balance coefficient of the loss term.

**Figure 7:**
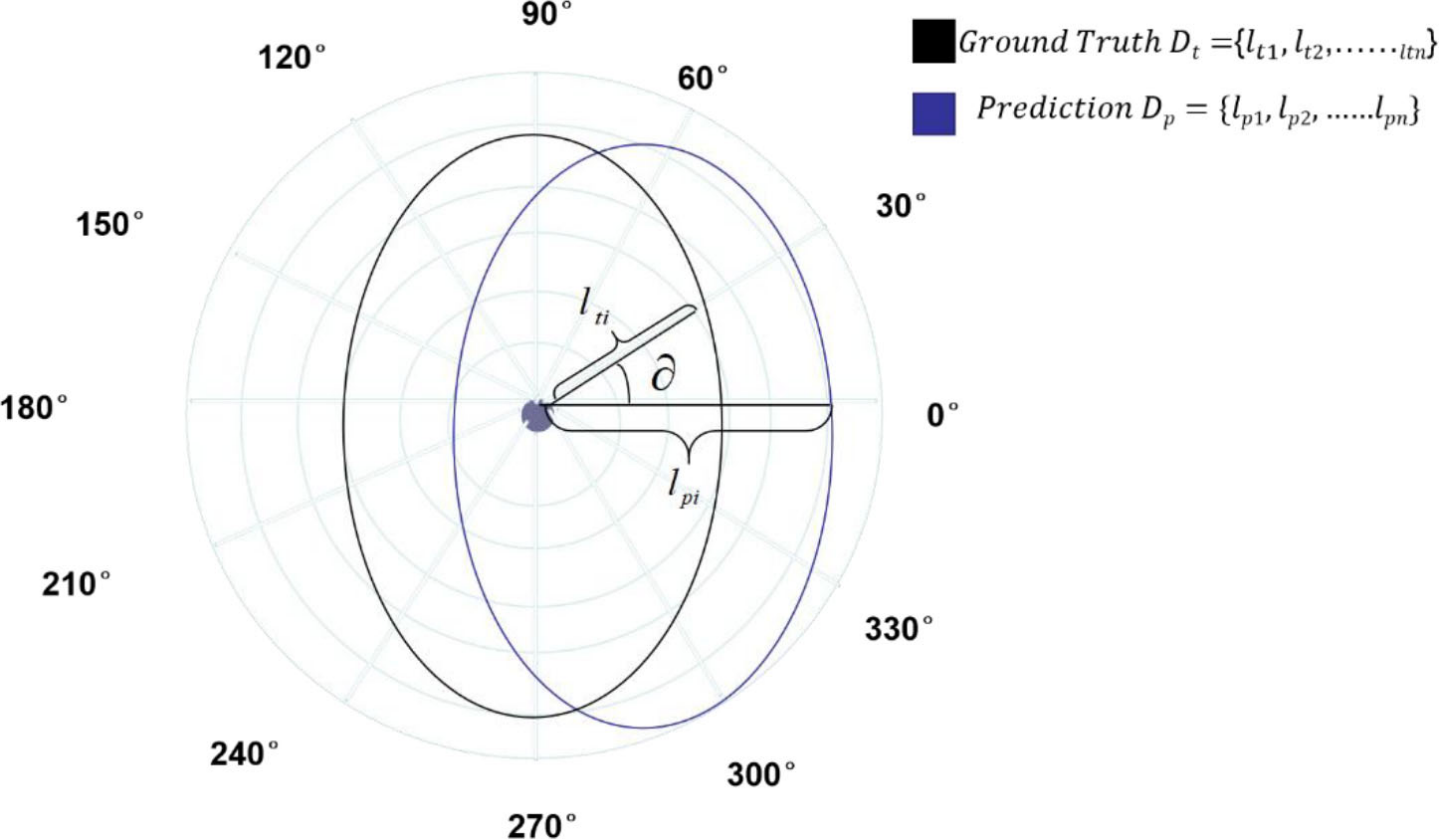
Polar IoU loss representation diagram.

The mask branch in the Polar-Net model is mainly used to realize the regression of dense distances. When actually building the regression model, the following two problems are likely to arise: first, for the regression of the distance from the centroid to the outline of the instance actually regresses the length of n rays (n=36), which is still relatively dense compared to the classification part, thus causing an imbalance between the regression loss and the classification loss. Second, for instance, the 36 rays to be regressed should all be regressed as a whole, rather than individually. In order to better solve the regression problem of this model, Polar IoU loss is introduced in the Polar-Net model based on IoU loss, and the area intersection ratio (IOU) between the predicted area and the true value area is calculated in polar coordinates, as shown in **Figure 7**. That is:


(12) $$IoU=\frac{\int □\overset{2\pi }{\underset{0}{\;}}\frac{1}{2}min{\left(l,l*\right)}^{2}d\partial }{\int □\overset{2\pi }{\underset{0}{\;}}\frac{1}{2}max{\left(l,l*\right)}^{2}d\partial }$$

Here the regression target *l* and the predicted *l* ^*^ are the lengths of the rays.

This equation is then converted to the discrete state as:


(13) $$l_{min□}=min(l,l^{*}),l_{max□}=(l,l^{*})$$

(14) $$IoU=\underset{N\to \infty }{\lim}\frac{\sum_{i=1}^{N}\frac{1}{2}l_{\min}^{2}\Delta \partial_{i}}{\sum_{i=1}^{N}l_{\max}^{2}\Delta \partial_{i}}$$

Then if the power form is discarded and simplified to the following form, the impact on performance is minimal. The final Polar Iou Loss is a binary cross-entropy loss. Since the optimal IoU is always one, the loss is actually the negative logarithm of Polar Iou:


(15) $$\text{Polar IoU Loss }=\;log\frac{\sum_{i=1}^{n\sum }l_{max}}{\sum_{i=1}^{n\sum }l_{min}}$$

The loss function proposed here for the regression problem can solve the 36-ray overall regression problem on the one hand, and on the other hand it is differentiable, can be back-propagated, and is very easy to implement parallel computation, thus facilitating a fast-training process for overall prediction of regression metrics. By conducting experimental comparisons with other loss functions, it is shown that the method improves the overall performance of the system and is able to automatically maintain a balance between classification loss and regression loss for dense distance prediction.

## 4 Results and analysis

### 4.1 Experimental design and parameter setting

The processing platform for both the experimental algorithms and their comparison algorithms are run on a server equipped with Ubuntu 18.04LTS operating system, 32GB GPU Tesla V100 and V10.0 CUDA environment, and all programs are written in Python language and based on Pytorch 1.4 deep learning library.

Migration learning can solve the overfitting problem of the model in the training process; therefore, we use a pre-trained model based on the COCO dataset as the initial weight model for this experiment, based on which the loss function can converge to a stable value as soon as possible and speed up the training data. The model is optimized using stochastic gradient descent stochastic gradient descent (SGD) (Bottou, 2010), and we set the initial learning rate to 0.0025, the weight decay to 0.0001, and the momentum of 0.9. The maximum number of iterations is 24 when performing algorithm training, and the model is stored once per iteration. The goodness of the model is not better with more iterations, the training accuracy of the model increases rapidly with the number of iterations and is generally stable. Too much training may lead to overfitting, so the trained model needs to be tested and assessed.

### 4.2 Model training

The efficient instance segmentation optimization model for green target fruit proposed in this study is as follows. Rich green fruit images are collected in an orchard environment using a Canon EOS 80D DSLR camera. Compress captured green fruit images and reduce the image resolution from 6000×to 4000 to 600×400. Images were labeled using LabelMe software, and each target fruit was labeled as an independent connected domain to produce a COCO format dataset. Use the produced dataset as the input of the cavernous convolutional network. Build the Polar-Net base network framework and analyze the network structure. Polar-Net base network framework, get the optimal network training model, and train the input data to get the green fruit segmentation model. Input test to evaluate the segmentation results of the obtained green fruit segmentation model utilizing evaluation metrics, and adjust the parameters of the model according to the evaluation structure. Repeat the training to improve the model until the optimal network model is generated.

### 4.3 Evaluation metrics

In order to evaluate the effectiveness of this model for green spherical fruit recognition segmentation, two indicators, accuracy and running time, are used to evaluate the model in this paper, and the values of accuracy are both in the range of [0,1], where Averagetime is defined as the evaluation indicator of average running time, as follows.


(16) $$Precision=\frac{TP}{TP+FP}$$

(17) $$Averagetime=\frac{\sum_{i=1}^{n}t}{n}$$

Where TP is the number of true positive samples, FP is the number of false positive samples, t is the time required for each iteration, and n is the number of iterations.

### 4.4 Green target fruit splitting effect

**Figure 8:**
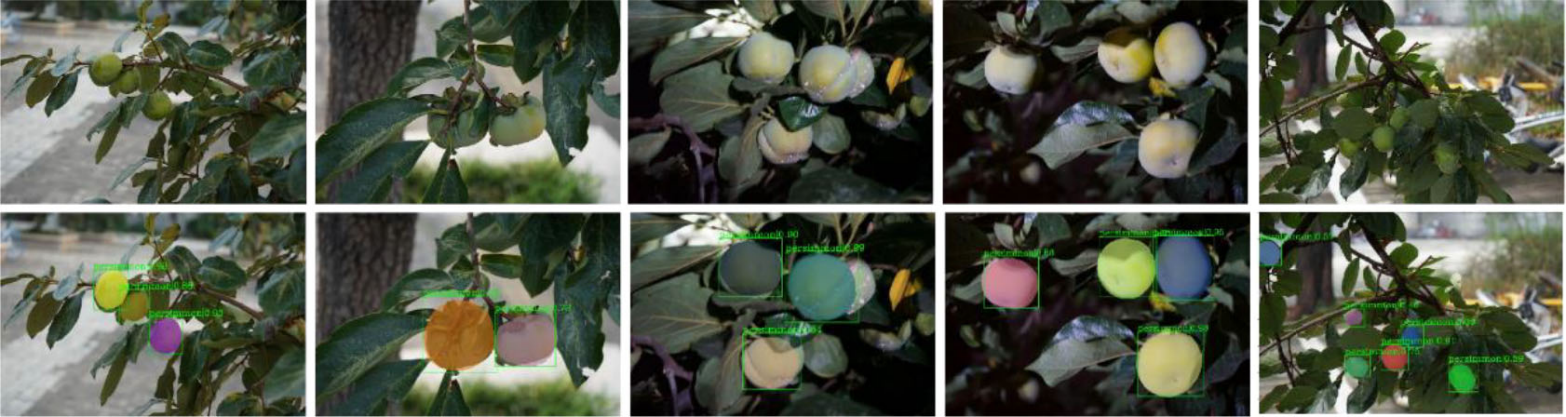
Effect of segmentation on unripe persimmon(The first row represents the original data set, and the second row shows the effect after segmentation by Polar-Net model).

In real orchard scenes, the target fruit is not the same in the images, and the segmentation effect will be affected to different degrees. For example, in images with sparse fruits, the recognition will be clearer and more obvious; but for those overlapping, same color images, it will not be easy to segment in the actual segmentation process; at night or in images with backlight or water drops, it is also difficult for segmentation. In this study, the Polar-Net instance segmentation model is used to segment the green unripe persimmons, and the segmentation effect is shown in **Figure 8**.

As can be seen in **Figure 8**, we acquired persimmon images with denser fruits, a larger number of fruits, the presence of a situation where they are heavily obscured by leaves and branches, a small target fruit sampled in the distant view, a more complex image acquisition environment, and persimmon fruit images in a variety of complex environments were selected. The performance of this optimization model is evaluated for these images in complex environments for fruit segmentation, selection accuracy and time required.

Since the model is based on detection followed by segmentation, to demonstrate its performance, the accuracy for detection and segmentation is recorded separately, and **Table 2** below shows the accuracy of segmentation and detection of Polar-Net after testing on the Persimmon sub-dataset.

**Table 2:** Accuracy of detection and segmentation under the data set.

Detection		Segmentation	
Metric	Value	Metric	Value
*mAP^b^*	69.8%	*mAP^s^*	65.1%
$AP_{50}^{b}$	89.9%	$AP_{50}^{s}$	88.7%
$AP_{75}^{b}$	76.6%	$AP_{75}^{s}$	71.9%
$mAP_{s}^{b}$	22.9%	$mAP_{s}^{s}$	13.6%
$mAP_{m}^{b}$	68.9%	$mAP_{m}^{s}$	64.5%
$mAP_{l}^{b}$	81.5%	$mAP_{l}^{s}$	79.7%

where, $AP_{50}^{b}\;,\;AP_{50}^{s}$ are the accuracy of the model’s prediction borders and mask segmentation under the threshold of IoU=0.5; *mAP^mb^*, *mAP_□_^ms^* are the accuracy of the model’s prediction borders and prediction masks for the network under 10 IoU thresholds of [0.5, 0.55, 0.6,…, 0.95] and averaging the 10 APs obtained; furthermore, $mAP_{\bar{s}}\;,\;mAP_{\bar{m}}$ , $mAP_{\bar{l}}$ are the combined results of the model for small-scale fruit, medium-scale fruit, and large-scale fruit prediction results in the three scales of [0,322], [322,962], and [962,INF], respectively.

#### 4.4.1 Algorithm comparison

To further illustrate the effectiveness of the model for green fruit recognition segmentation, the current advanced algorithms for target detection and segmentation were selected from two performance perspectives, respectively, including anchor-frame-based two-stage algorithm: Faster R-CNN; anchor-frame-based single-stage algorithms: SSD512 (Liu et al., 2016), YOLACT (Bolya et al., 2019), SOLO (Wang et al., 2020) and single-stage algorithms without anchor frames: FCOS, FoveaBox (Kong et al., 2020) and the original PolarMask method comparison algorithm with Polar-Net model using the same persistence sub-dataset, on the same configuration of the experimental platform, the initial learning rate are set to 0.0025, the weight decay are set to 0.0001, both set the momentum to 0.9, run and evaluate the algorithm performance in detecting or segmenting fruits under complex natural conditions. When evaluating the algorithm, in addition to assessing the accuracy of the algorithm’s detection or segmentation, we also need to consider the time the algorithm takes to actually detect or segment the target. We are able to reduce the detection or segmentation time while ensuring accuracy, and if the segmentation time does not meet the real-time requirements to be truly operational, it loses its relevance. **Table 3** below lists the differences in recognition performance between the comparison algorithms and Polar-Net, and the specific experimental evaluation results are presented in **Table 3**.

**Table 3:** Recognition detection segmentation effect of each model on persimmon data set.

methods	*mAP^b^*	*Averagetime^b^*	mAP^s^	*Averagetime^s^*
*Two-stage anchor-based*				
Faster RCNN	70.0%	0.50s	**—**	**—**
*One-stage anchor-based*				
YOLACT	64.7%	0.46s	61.0%	0.45s
SSD512	64.1%	0.85s	**—**	**—**
SOLO	**—**	**—**	58.6%	0.49s
*One-stage anchor-free*				
FCOS	66.0%	1.19s	**—**	**—**
Foveabox	68.1%	1.24s	**—**	**—**
PolarMask	66.4%	0.44s	63.6%	0.52s
ours	69.8%	0.41s	65.1%	0.44s

where *mAP^b^* indicates the detection accuracy, *mAP^s^* indicates the segmentation accuracy, *Averagetime^b^* indicates the time required by detecting, *Averagetime^b^* indicates the time required by segmentation, and “-” indicates that the model does not have the ability to predict borders or masks, for example, Faster R-CNN is a target detection algorithm, which can only predict the border to locate the target fruit in the image, but cannot segment the fruit mask, so the value of *mAP^s^* is “-”; SOLO is an instance segmentation algorithm, but its segmentation does not need to rely on the border predicted by the detector, but to segment each fruit directly Therefore, the value of *mAP^b^* is “-”.

In terms of detection performance, the method in this paper has further improved on PolarMask and has good performance in segmentation performance. In the comparison of many algorithms, the Polar-Net method clearly achieves better results in the evaluation on the persistence sub-dataset and is only slightly inferior to Faster runs in detection, but both in terms of model capacity, complexity and detection speed, Polar-Net makes up for its small loss in accuracy with a cleaner architecture, less computation and faster detection speed. Polar-Net, on the other hand, is more efficient in detection, takes less time, and further improves the operation speed while ensuring accuracy. Therefore, after the above analysis, our method achieves excellent results in terms of both segmentation accuracy and segmentation time, and can basically meet the real-time requirements with strong generalization ability and robustness.

## 5 Conclusion

In the real complex unstructured orchard environment, for the challenge of efficient segmentation of green target fruit, this study proposes Polar-Net green target fruit instance segmentation model with unripe green persimmon as the research object. The new model uses are DenseNet as the backbone network for feature extraction, and introduce FPN, CFN, and RPN structures to achieve different scale feature extraction and obtain ROI regions as the input of Polar-Net head network. The target centroids are found in polar branch and polar coordinates are completed to establish, and the target fruit contours are found together with mask branch to complete the instance segmentation of the target fruit. During the training process, Polar IoU loss is used for the loss function. Testing the model on both the persimmon dataset and coco dataset showed substantial improvement in accuracy over existing model algorithms. The Polar-Net model achieves accurate segmentation of green fruit in complex environments with its simple model architecture and less computational storage.

The Polar-Net model achieves accurate detection and segmentation of green fruit in complex environments, and the segmentation speed is greatly improved. Moreover, the model is a single-stage anchoress frame, and its generalization ability is strong, so the model can be applied to the production detection of other fruit and vegetables. In future research, the complexity of the orchard environment needs to be further considered, and the model needs to be further improved for the existence of force major such as shading in the orchard environment to improve the real-time working ability of the model, so that it can be better applied to work in the miscellaneous orchard environment.

## Data availability statement

The raw data supporting the conclusions of this article will be made available by the authors, without undue reservation.

## Author contributions

WJ: conceptualization, writing—original draft preparation, and funding acquisition. JL: data curation, software, and writing—original draft preparation. QL: methodology, software, and validation. QL: data curation and visualization. TZ: data curation and software. XD: conceptualization, funding acquisition, and writing—reviewing and editing. All authors contributed to the article and approved the submitted version.
